# Prevalence of Impairing Substance Use in Injured Drivers

**DOI:** 10.1001/jamanetworkopen.2025.6379

**Published:** 2025-04-22

**Authors:** Jeffrey R. Brubacher, Shannon Erdelyi, Herbert Chan, Sarah Simmons, Paul Atkinson, Floyd Besserer, David B. Clarke, Phil Davis, Raoul Daoust, Marcel Émond, Jeff Eppler, Jacques S. Lee, Andrew MacPherson, Kirk Magee, Eric Mercier, Robert Ohle, Mike Parsons, Jagadish Rao, Brian H. Rowe, John Taylor, Christian Vaillancourt, Ian Wishart

**Affiliations:** 1 Department of Emergency Medicine, The University of British Columbia, Vancouver, British Columbia, Canada; 2 Department of Emergency Medicine, Dalhousie Medicine, Saint John, New Brunswick, Canada; 3 Division of Medical Sciences, University of Northern British Columbia, Prince George, British Columbia, Canada; 4 Department of Surgery, Division of Neurosurgery, Dalhousie University, Halifax, Nova Scotia, Canada; 5 Department of Emergency Medicine, University of Saskatchewan, Saskatoon, Saskatchewan, Canada; 6 Department of Emergency and Family Medicine, University of Montreal, Montreal, Quebec, Canada; 7 Department of Family and Emergency Medicine, Université Laval, Quebec City, Quebec, Canada; 8 Department of Emergency Medicine, University of Toronto, Toronto, Ontario, Canada; 9 Department of Emergency Medicine, Dalhousie University, Dalhousie University, Halifax, Nova Scotia, Canada; 10 Department of Emergency Medicine, Northern Ontario School of Medicine, Thunder Bay, Ontario, Canada; 11 Department of Emergency Medicine, Memorial University, St John’s, New Foundland and Labrador, Canada; 12 Department of Surgery, University of Saskatchewan, Regina, Saskatchewan, Canada; 13 Department of Emergency Medicine, University of Alberta, Edmonton, Alberta, Canada; 14 School of Public Health, University of Alberta, Edmonton, Alberta, Canada; 15 Department of Emergency Medicine, University of Ottawa, Ottawa, Ontario, Canada; 16 Department of Emergency Medicine, University of Calgary, Calgary, Alberta, Canada

## Abstract

**Importance:**

Impaired driving is an important public health issue, but its prevalence is challenging to monitor.

**Objectives:**

To report the prevalence of alcohol, cannabis, recreational drugs, and sedating medications in injured Canadian drivers, identify demographic and collision factors associated with drug or alcohol use, and compare the prevalence of drug-involved driving in different parts of Canada.

**Design, Setting, and Participants:**

This cross-sectional study prospectively obtained blood samples from injured drivers treated in 15 Canadian trauma centers and measured blood levels of tetrahydrocannabinol (THC; the main impairing compound in cannabis), alcohol, stimulants, opioids, and depressants from January 2019 to June 2023. Data were analyzed from April to May 2024.

**Exposure:**

Blood levels of THC, alcohol, stimulants, opioids, and depressants.

**Main Outcomes and Measures:**

Demographic and collision details were extracted from medical records. The crude prevalence for each substance class among all injured drivers and in selected subgroups was computed. Logistic regression models identified factors associated with substance use.

**Results:**

Of 8328 injured drivers (mean [SD] age, 43 [18] years; median [IQR] age, 40 [28-57] years; 5605 male [67.3%]; 2723 female [32.7%]), 4568 (54.9%) tested positive for an impairing substance and 1798 (21.6%) tested positive for 2 or more substance classes. Depressants, as a class, were detected in 2368 drivers (28.4%). THC was the most commonly detected single substance (1354 drivers [16.3%]), followed by alcohol (1341 drivers [16.1%]). Stimulants (1057 drivers [12.7%]) and opioids (905 drivers [10.9%]) were also detected. Substances were detected less often in drivers aged 75 years or older (195 of 455 drivers [42.9%]) and younger than 19 years (149 of 304 drivers [49.0%]). THC was most common in drivers aged 19 to 24 years, alcohol in drivers aged 19 to 34 years, stimulants in drivers aged 35 to 44 years, opioids in drivers aged 55 to 64 years, and depressants in drivers aged 65 to 74 years. Males had similar prevalence of substance use as females (3141 males [56.0%] vs 1427 females [52.4%]); more males used alcohol (adjusted odds ratio [aOR], 1.53; 95% CI, 1.21-1.92), cannabis (aOR, 1.66; 95% CI, 1.48-1.86), and stimulants (aOR, 1.53; 95% CI, 1.34-1.75), but males were less likely to have used a depressant (aOR, 0.54; 95% CI, 0.47-0.62). Rural drivers were more likely to use alcohol (aOR, 1.51; 95% CI, 1.29-1.76), stimulants (aOR, 1.32; 95 CI, 1.03-1.70), depressants (aOR, 1.28; 95% CI, 1.09-1.51), opioids (aOR, 1.26; 95% CI, 1.08-1.47), any substance (aOR, 1.40; 95% CI, 1.20-1.63), or multiple classes of substances (aOR, 1.55; 95 CI, 1.23-1.95). There was substantial geographic variation in the prevalence of substance use in injured drivers.

**Conclusions and Relevance:**

These findings suggest that impaired driving is a substantial road safety concern in Canada. Continued monitoring is required to develop targeted interventions and to evaluate the effectiveness of prevention measures.

## Key Points

**Question:**

What is the prevalence of substance use in injured drivers in Canada?

**Findings:**

In a sample of 8328 drivers treated in a Canadian trauma center after a collision, more than one-half (54.9%) tested positive for an impairing substance, including depressants (28.4%), tetrahydrocannabinol (16.3%), alcohol (16.1%), stimulants (12.7%), and opioids (10.9%). Prevalence varied by age, sex, rurality, collision type, and region.

**Meaning:**

These findings suggest that impaired driving is a substantial road safety concern and that continued monitoring is required to develop targeted interventions and to evaluate the effectiveness of prevention measures.

## Teaser

This cross-sectional study assesses the prevalence of alcohol, cannabis, recreational drugs, and sedating medications in injured drivers in Canada and identifies demographic and collision factors associated with substance use.

## Introduction

Globally, 1.2 million people die from road trauma each year and many more are injured.^1^ Many of these collisions are caused by impaired driving. Alcohol-impaired driving is well known to cause collisions.^2^ Cannabis-impaired driving has attracted increased attention due to global trends toward cannabis legalization and is also associated with increased crash risk.^3^ Depressants, opioids, and stimulants also increase collision risk.^4,5,6,7^

Monitoring drug-involved driving is difficult, and prevalence estimates may be biased by methodological limitations. Drug testing requires collection and analysis of biological fluids, such as urine, saliva, or blood, which can be difficult to obtain and have varying degrees of diagnostic accuracy. Furthermore, unbiased prevalence estimates require random selection and standardized testing. The major sources used to monitor drug-involved driving in North America—roadside surveys, coroner data, and police or hospital data from crash-involved drivers—likely misrepresent the true prevalence. Roadside surveys typically obtain saliva samples from consenting drivers and analyze them for drugs in a toxicology laboratory. This approach is limited by refusal rates of 20% to 30%, which may bias results.^8,9^ Further, blood testing is the benchmark for detecting drugs, and drug concentrations in saliva correlate poorly with those in blood or with impairment.^10^ Coroner data may be susceptible to bias if drug testing is based on suspicion of drug use and/or racial or gender bias. There may also be differences in drug concentration between the time of collision and when coroner samples are obtained due to postmortem distribution.^11^ Drivers who have been in a collision may be drug tested by police as part of the collision investigation or in hospital as part of clinical management. These approaches may be susceptible to selection bias if testing is based on suspicion of drug use. The objectives of this study are to (1) report the prevalence of alcohol, cannabis, recreational drugs, and sedating medications in injured drivers in Canada; (2) identify demographic and collision factors associated with increased prevalence for drug or alcohol use; and (3) compare the prevalence of drug-involved driving in different parts of the country.

## Methods

This cross-sectional study was approved by the research ethics boards of participating institutions with waivers of informed consent because procedures to protect personal information were observed and because blood samples analyzed were only those that were excess from clinical use. The reporting of the study followed the Strengthening the Reporting of Observational Studies in Epidemiology (STROBE) reporting guideline.

### Setting

Participating emergency departments (EDs) operate within regional trauma centers from 15 cities in 8 Canadian provinces. This study is restricted to data collected when all 8 provinces were participating (January 2019 to June 2023); a total of 15 trauma centers participated. According to the 2021 National Census, the total census subdivision of these cities is 10.5 million, which is more than one-quarter of the entire Canadian population (38.25 million).^12^

### Design

Methods of this cross-sectional study have been published previously.^13,14,15^ They are summarized below.

### Population

We prospectively identified drivers who were assessed in the ED for injuries following a motor vehicle crash. Drivers of on-road vehicles (automobiles, sport-utility vehicles, pickup trucks, vans, heavy vehicles, and motorcycles) who had blood obtained within 6 hours of a collision were eligible. Drivers who died at the scene or in the ED were excluded because their medical records and blood samples were inconsistently available across hospital sites due to privacy and legal concerns. Drivers with minor injuries who did not require bloodwork were excluded.

### Procedures

Nonstudy clinicians at participating hospitals obtained blood samples to guide trauma management when there was evidence that the driver may have had moderate to severe injuries based on mechanism of injury (eg, high-speed collision) and/or clinical evidence that the driver was injured (eg, unstable vital signs). Research assistants identified eligible drivers and obtained blood that remained after clinical use before it was discarded by hospital laboratories. Cases with no excess blood available were excluded. Blood was frozen for later toxicology analysis at a central laboratory. Research assistants, trained by the project manager (H.C.), reviewed medical records of all eligible drivers and used standardized forms to record basic demographic, medical, and collision information.

### Toxicology

Broad-spectrum toxicology testing for cannabinoids, other recreational drugs (cocaine, amphetamines, and opiates), and psychotropic pharmaceuticals (including antihistamines, benzodiazepines, other hypnotics, and sedating antidepressants) was done with liquid chromatography-tandem mass spectrometry. Alcohol level was measured with gas chromatography. Detection limits were 0.2 ng/mL for tetrahydrocannabinol (THC; the primary psychoactive ingredient in cannabis), 0.01% for alcohol, and 1 ng/mL for other drugs. In most cases, samples consisted of whole blood. When only plasma specimens were available, results were adjusted to equivalent whole blood results.^16^ We reported the following substance categories: THC detectable, THC greater than 5 ng/mL, blood alcohol concentration (BAC) detectable, BAC greater than 0.08%, stimulants detectable, depressants detectable, and opioids detectable. eTable 1 in Supplement 1 shows the substances included in each category. Note that a BAC of 0.08% (80 mg/dL or 17.3 mmol/L) is the legal concentration for operating a motor vehicle in Canada. For cannabis, it is illegal to drive with blood THC greater than 2 ng/mL with higher penalties for THC greater than 5 ng/mL.

### Risk Factors

We measured the association of risk factors with substance detection. These included sex, age range, residential postal code (urban or rural based on Canada Post designation),^17^ time of crash (4-hour blocks), day of crash, year, season, crash type (single-vehicle or multivehicle), injury severity (admitted to hospital or discharged from the ED), and region (British Columbia, Alberta, Saskatchewan, Ontario, Quebec, and the Atlantic provinces).

### Statistical Analysis

We computed the crude prevalence for each substance (or substance class) among all included drivers and in selected subgroups of drivers (objective 1). To identify factors associated with substance use (objective 2), we fit separate logistic regression models with each substance of interest as the outcome. Models included all risk factors mentioned previously as covariates. Given the low rate of missing covariates (3.7%), drivers with missing values were excluded. We used cluster-robust standard errors with small-sample correction (CR2 estimator) to account for correlation between drivers who visited the same hospital site.^18^ Because risk factors are categorical, often with more than 2 possible values, we used Wald χ^2^ tests to compute *P* values for the overall effect of each risk factor. Unlike likelihood ratio tests, Wald tests do not require the assumption of independence within clusters. We report adjusted odds ratios (aORs) and 95% CIs derived from model coefficients. Because analyses were exploratory, we considered associations with each factor and substance threshold as separate hypotheses. *P* values less than .05 were considered statistically significant.^19^ To examine geographic variation in substance use (objective 3), we computed crude prevalence in each region with 95% CIs derived from 1-sample proportions assuming asymptotic normality. Statistical analysis was conducted from April to May 2024 using R version 4.3.1 (R Project for Statistical Computing).

## Results

During the study period, 12 486 drivers had blood tests ordered; of these, 2381 were excluded because blood was taken more than 6 hours after the collision, 1772 were excluded because excess blood was unavailable, and 5 were excluded because they died in the ED. In total, 8328 drivers (mean [SD] age, 43 [18] years; median [IQR] age, 40 [28-57] years; 5605 male [67.3%]; 2723 female [32.7%]) were included (Table 1). Of these, 6916 (83.0%) had an urban address and 3039 (36.5%) required hospital admission. Two-thirds of collisions (5369 collisions [64.5%]) occurred during a weekday, 5485 (65.8%) were between 10 am and 10 pm, and 3373 (40.5%) were single-vehicle collisions. eTable 2 in Supplement 1 provides additional information on the drivers and associated collisions.

**Table 1.** Characteristics of Injured Drivers and Collisions by Region

Characteristic	Injured drivers, No. (%)						
	National (N = 8328)	British Columbia (n = 2418)	Alberta (n = 1605)	Saskatchewan (n = 577)	Ontario (n = 1823)	Quebec (n =1281)	Atlantic Provinces (n = 624)
Age group, y							
<19	304 (3.7)	54 (2.2)	79 (4.9)	37 (6.4)	45 (2.5)	55 (4.3)	34 (5.4)
19-24	1068 (12.8)	247 (10.2)	210 (13.1)	100 (17.3)	261 (14.3)	162 (12.6)	88 (14.1)
25-34	1874 (22.5)	564 (23.3)	310 (19.3)	127 (22.0)	411 (22.5)	311 (24.3)	151 (24.2)
35-44	1445 (17.4)	422 (17.5)	326 (20.3)	87 (15.1)	302 (16.6)	208 (16.2)	100 (16.0)
45-54	1243 (14.9)	387 (16.0)	240 (15.0)	69 (12.0)	285 (15.6)	166 (13.0)	96 (15.4)
55-64	1223 (14.7)	376 (15.6)	235 (14.6)	75 (13.0)	251 (13.8)	200 (15.6)	86 (13.8)
65-74	716 (8.6)	233 (9.6)	119 (7.4)	56 (9.7)	157 (8.6)	107 (8.4)	44 (7.1)
>74	455 (5.5)	135 (5.6)	86 (5.4)	26 (4.5)	111 (6.1)	72 (5.6)	25 (4.0)
Sex							
Male	5605 (67.3)	1596 (66.0)	1038 (64.7)	393 (68.1)	1325 (72.7)	827 (64.6)	426 (68.3)
Female	2723 (32.7)	822 (34.0)	567 (35.3)	184 (31.9)	498 (27.3)	454 (35.4)	198 (31.7)
Residential postal code							
Urban	6916 (83.0)	2285 (94.5)	1348 (84.0)	261 (45.2)	1434 (78.7)	1071 (83.6)	517 (82.9)
Rural	1161 (13.9)	87 (3.6)	197 (12.3)	225 (39.0)	357 (19.6)	193 (15.1)	102 (16.3)
Missing	251 (3.0)	46 (1.9)	60 (3.7)	91 (15.8)	32 (1.8)	17 (1.3)	5 (0.8)
Injury severity							
Admitted	3039 (36.5)	606 (25.1)	490 (30.5)	233 (40.4)	979 (53.7)	481 (37.5)	250 (40.1)
Treated and released	5280 (63.4)	1811 (74.9)	1115 (69.5)	342 (59.3)	843 (46.2)	795 (62.1)	374 (59.9)
Missing	9 (0.1)	1 (<0.1)	0	2 (0.3)	1 (0.1)	5 (0.4)	0
Crash type							
Single-vehicle	3373 (40.5)	786 (32.5)	678 (42.2)	311 (53.9)	711 (39.0)	520 (40.6)	367 (58.8)
Multivehicle	4953 (59.5)	1632 (67.5)	927 (57.8)	266 (46.1)	1110 (60.9)	761 (59.4)	257 (41.2)
Missing	2 (<0.1)	0	0	0	2 (0.1)	0	0
Time between crash and blood draw, min							
Mean (SD)	117 (71)	125 (66)	111 (67)	124 (69)	99 (72)	135 (77)	107 (68)
0-60	1667 (20.0)	246 (10.2)	353 (22.0)	66 (11.4)	675 (37.0)	182 (14.2)	145 (23.2)
61-120	2277 (27.3)	792 (32.8)	424 (26.4)	163 (28.2)	302 (16.6)	445 (34.7)	151 (24.2)
121-240	3733 (44.8)	1195 (49.4)	729 (45.4)	296 (51.3)	714 (39.2)	514 (40.1)	285 (45.7)
240-360	651 (7.8)	185 (7.7)	99 (6.2)	52 (9.0)	132 (7.2)	140 (10.9)	43 (6.9)

**Figure 1.**
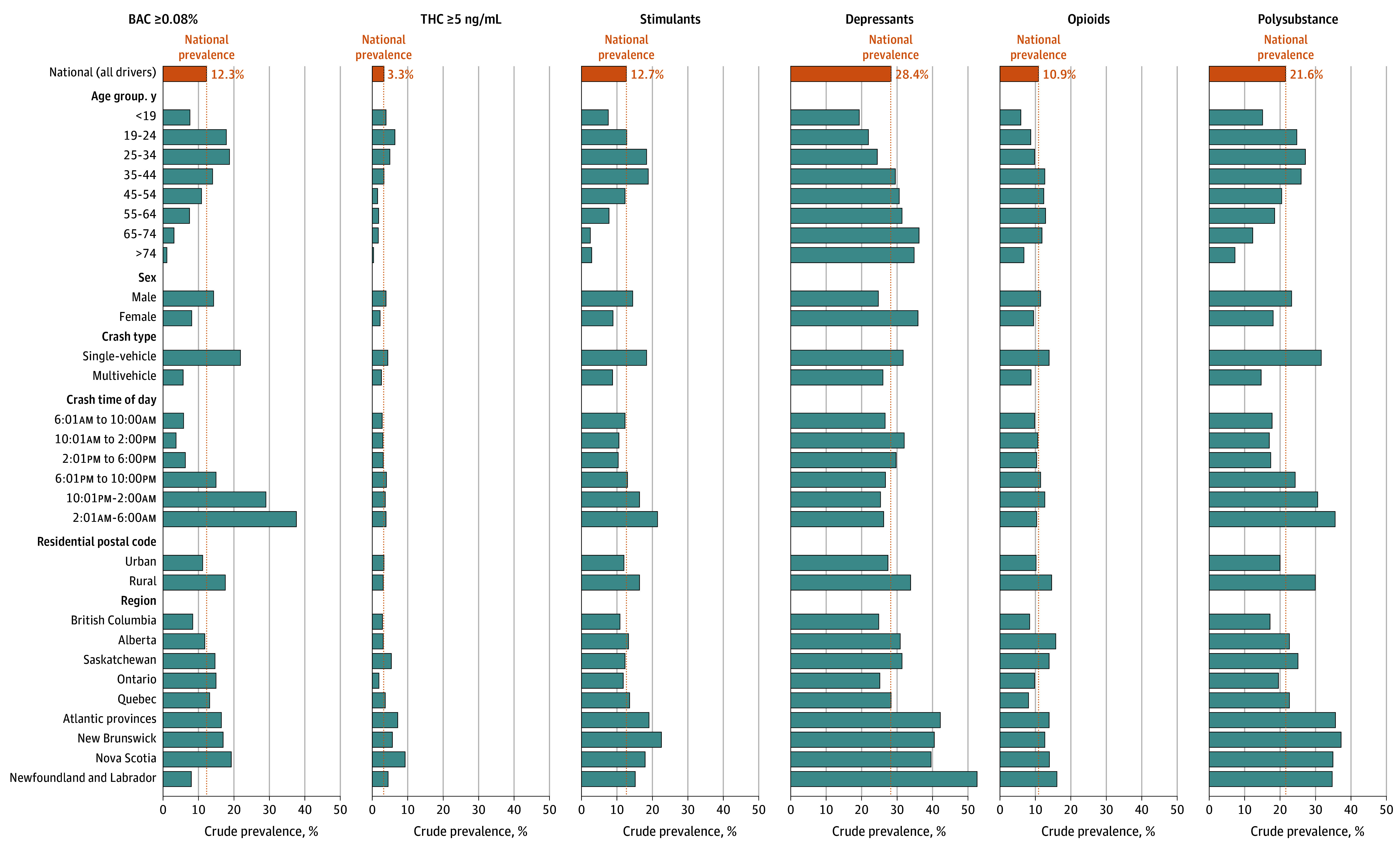
Crude Prevalence of Substances and Substance Classes Among Injured Drivers for Select Risk Factors BAC indicates blood alcohol concentration; THC, tetrahydrocannabinol.

Table 2, eTable 3 in Supplement 1, and Figure 1 show toxicology findings according to risk factor. Of all drivers, 4568 (54.9%) tested positive for an impairing substance, including 1341 (16.1%) with BAC greater than 0%, 1354 (16.3%) with THC greater than 0 ng/mL, 1057 (12.7%) with stimulants, 905 (10.9%) with opioids, and 2368 (28.4%) with depressants. One in 8 drivers (1024 drivers [12.3%]) had BAC greater than 0.08% and 277 (3.3%) had THC greater than 5 ng/mL. One in 5 drivers (1798 drivers [21.6%]) had used more than 1 class of substance. Substances were least commonly detected in drivers aged 75 years or older (195 of 455 drivers [42.9%]) followed by drivers younger than 19 years (149 of 304 drivers [49.0%]) and drivers aged 65 to 74 years (352 of 716 drivers [49.2%]). Substances were detected in 3141 males (56.0%) and 1427 females (52.4%). Males had higher prevalence of alcohol, THC, stimulants, and opioids, and females had a higher prevalence of depressants.

**Table 2.** Substance Prevalence Among Injured Drivers by Select Risk Factors

Factor	Injured drivers, No./total No. (%)									
	BAC >0	BAC ≥0.08%	THC >0	THC ≥5 ng/mL	Stimulants	Depressants		Opioids	Any substance	Polysubstance
National (all drivers)	1341/8328 (16.1)	1024/8328 (12.3)	1354/8328 (16.3)	277/8328 (3.3)	1057/8328 (12.7)		2368/8328 (28.4)	905/8328 (10.9)	4568/8328 (54.9)	1798/8328 (21.6)
Age group, y										
<19	42/304 (13.8)	23/304 (7.6)	64/304 (21.1)	12/304 (3.9)	23/304(7.6)		59/304 (19.4)	18/304 (5.9)	149/304 (49.0)	46/304 (15.1)
19-24	243/1068 (22.8)	191/1068 (17.9)	294/1068 (27.5)	68/1068 (6.4)	137/1068 (12.8)		235/1068 (22.0)	93/1068 (8.7)	625/1068 (58.5)	265/1068 (24.8)
25-34	434/1874 (23.2)	353/1874 (18.8)	438/1874 (23.4)	94/1874 (5.0)	344/1874 (18.4)		460/1874 (24.5)	183/1874 (9.8)	1132/1874 (60.4)	509/1874 (27.2)
35-44	252/1445 (17.4)	202/1445 (14.0)	222/1445 (15.4)	48/1445 (3.3)	273/1445 (18.9)		428/1445 (29.6)	183/1445 (12.7)	849/1445 (58.8)	376/1445 (26.0)
45-54	175/1243 (14.1)	136/1243 (10.9)	147/1243 (11.8)	19/1243 (1.5)	153/1243 (12.3)		382/1243 (30.7)	154/1243 (12.4)	648/1243 (52.1)	255/1243 (20.5)
55-64	134/1223 (11.0)	92/1223 (7.5)	132/1223 (10.8)	22/1223 (1.8)	96/1223 (7.8)		385/1223 (31.5)	158/1223 (12.9)	618/1223 (50.5)	226/1223 (18.5)
65-74	49/716 (6.8)	22/716 (3.1)	41/716 (5.7)	12/716 (1.7)	18/716 (2.5)		260/716 (36.3)	85/716 (11.9)	352/716 (49.2)	88/716 (12.3)
>74	12/455 (2.6)	5/455 (1.1)	16/455 (3.5)	2/455 (0.4)	13/455 (2.9)		159/455 (34.9)	31/455 (6.8)	195/455 (42.9)	33/455 (7.3)
Sex										
Male	1061/5605 (18.9)	803/5605 (14.3)	1034/5605 (18.4)	216/5605 (3.9)	814/5605 (14.5)		1388/5605 (24.8)	647/5605 (11.5)	3141/5605 (56.0)	1306/5605 (23.3)
Female	280/2723 (10.3)	221/2723 (8.1)	320/2723 (11.8)	61/2723 (2.2)	243/2723 (8.9)		980/2723 (36.0)	258/2723 (9.5)	1427/2723 (52.4)	492/2723 (18.1)
Residential postal code										
Urban	1012/6916 (14.6)	772/6916 (11.2)	1081/6916 (15.6)	227/6916 (3.3)	828/6916 (12.0)		1899/6916 (27.5)	704/6916 (10.2)	3657/6916 (52.9)	1380/6916 (20.0)
Rural	270/1161 (23.3)	204/1161 (17.6)	217/1161 (18.7)	36/1161 (3.1)	190/1161 (16.4)		393/1161 (33.9)	169/1161 (14.6)	747/1161 (64.3)	348/1161 (30.0)

The pattern varied by age. Cannabis was most common in drivers aged 19 to 24 years, alcohol in drivers aged 19 to 34 years, stimulants in drivers aged 35 to 44 years, opioids in those aged 55 to 64 years, and depressants in those aged 65 to 74 years. Substance use was more prevalent in drivers with a rural address (747 of 1161 drivers [64.3%]) vs an urban address (3657 of 6916 drivers [52.9%]), especially for alcohol (204 rural drivers [17.6%] had BAC >0.08% vs 772 urban drivers [11.2%]). Polysubstance use was also higher in rural drivers (348 drivers [30.0%]) than in urban drivers (1380 drivers [20.0%]).

eTable 4 in Supplement 1 and the maps in the eFigure in Supplement 1 show prevalence of different substances by region. Substance use was most prevalent in the Atlantic provinces where two-thirds of drivers (69.6%; 95% CI, 65.7%-73.1%) tested positive for an impairing substance, and lowest in British Columbia where one-half (48.5%; 95% CI, 46.5%-50.5%) tested positive. There were slight variations in this pattern for individual substances.

**Figure 2.**
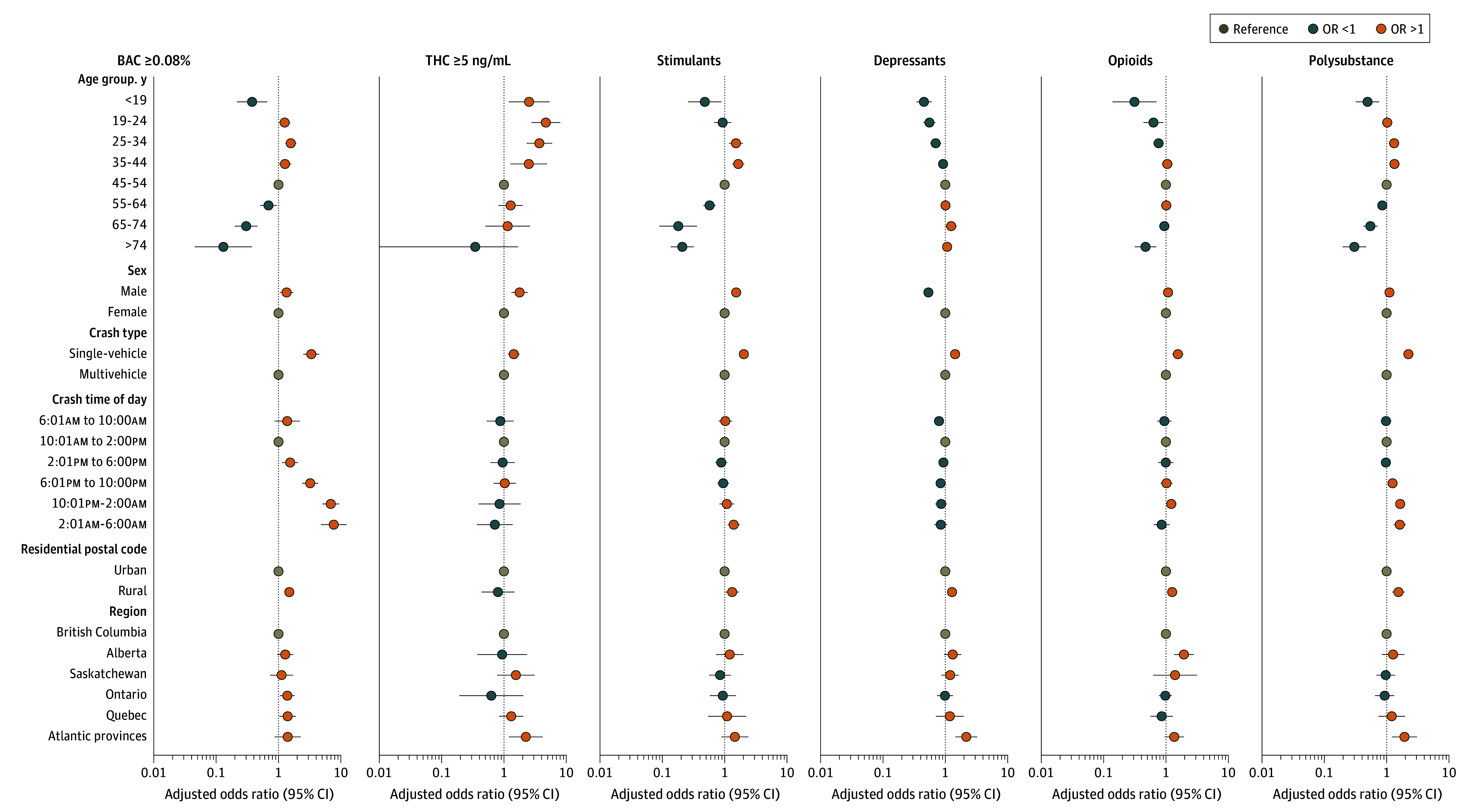
Adjusted Odds Ratios and 95% CIs for Select Risk Factors From Logistic Regression Models An odds ratio greater than 1 indicates higher risk of testing positive for the listed substance, while an odds ratio less than 1 indicates a lower risk of testing positive for the listed substance. BAC indicates blood alcohol concentration; THC, tetrahydrocannabinol.

eTable 5 in Supplement 1 and Figure 2 show results from our adjusted models. Compared with middle-aged drivers (ages 45-54 years), drivers younger than 45 years were more likely to test positive for THC (aORs ranging from 1.36; 95% CI, 1.07-1.73 to 2.79; 95% CI, 2.32-3.36) and drivers aged 19 to 44 years were more likely to have been drinking (aORs ranging from 1.25; 95% CI, 1.02-1.53 to 1.59; 95% CI, 1.33-1.90). Importantly, drivers younger than 19 years were less likely to have been drinking than middle-aged drivers (aOR, 0.58; 95% CI, 0.34-0.98). These models also show that males were more likely than females to have been drinking (aOR, 1.53; 95% CI, 1.21-1.92), to test positive for THC (aOR, 1.66; 95% CI, 1.48-1.86), or to have used a stimulant (aOR, 1.53; 95% CI, 1.34-1.75), but were less likely to have used a depressant (aOR, 0.54; 95% CI, 0.47-0.62).

Rural drivers were more likely to have been drinking (aOR, 1.51; 95% CI, 1.29-1.76) and more likely to have used stimulants (aOR, 1.32; 95 CI, 1.03-1.70), depressants (aOR, 1.28; 95% CI, 1.09-1.51), opioids (aOR, 1.26; 95% CI, 1.08-1.47), any substance (aOR, 1.40; 95% CI, 1.20-1.63), or multiple classes of substances (aOR, 1.55; 95 CI, 1.23-1.95). Many geographic differences were also significant. Compared with drivers from British Columbia, drivers from the Atlantic provinces were more likely to test positive for a depressant (aOR, 2.17; 95% CI, 1.45-3.26), for THC of 5 ng/mL or greater (aOR, 2.24; 95% CI, 1.21-4.18), for at least 1 impairing substance (aOR, 1.98; 95% CI, 1.30-3.02), or for multiple substances (aOR, 1.93; 95% CI, 1.22-3.07), and Alberta drivers were more likely to have used opioids (aOR, 1.94; 95% CI, 1.36-2.78).

## Discussion

This cross-sectional study reports toxicology results from 8328 injured drivers from 15 Canadian trauma centers. Although presence of an impairing substance does not necessarily mean the driver was impaired, our findings are concerning. More than one-half of drivers (54.9%) had used at least 1 impairing substance, and 1 in 5 (21.6%) had used 2 or more classes of substances, putting them at higher risk of collision.^7,20^ Depressants, including antihistamines, benzodiazepines and other hypnotics, antidepressants, anticonvulsants, and antipsychotics, were the most commonly detected class of impairing substances (28.4% of drivers). Depressants, especially benzodiazepines, increase collision risk.^4,7,21,22^ We also found that 12.7% of drivers tested positive for a stimulant and 10.9% had used an opioid. Stimulants and opioids also increase collision risk.^7,14^

Alcohol and THC were the 2 most commonly detected single substances. One in 6 drivers (16.1%) had been drinking alcohol, and 12.3% had BAC greater than 0.08%. Driving with BAC greater than 0.08% causes a 6-fold increase in collision risk^2^ and constitutes a criminal offense in Canada. THC was detected more often than alcohol (16.3%); however, detectable THC does not necessarily indicate recent cannabis use.^23^ Approximately 1 in 30 drivers (3.3%) had THC greater than 5 ng/mL. At a population level, drivers with THC greater than 5 ng/mL have increased risk of crashing, although this risk is less than for drivers with BAC greater than 0.08%.^7,14^ These statistics suggest that, although more drivers test positive for THC, alcohol remains the greater threat to road safety.

Research from other jurisdictions also indicates a high prevalence of impairing substances in injured drivers. Thomas et al^24^ studied 1856 injured drivers from 5 US trauma centers (2019-2020), and 56.0% tested positive for any impairing substance, including 24.2% for alcohol and 25.2% for THC. DiRago et al^25^ studied 4988 crash-involved drivers in Australia (2013-2018), and 38.4% tested positive for any impairing substance, 15.8% for alcohol, and 11.1% for THC. Legrand et al^26^ investigated 2492 injured drivers from Europe (2007-2010), and 33.3% tested positive for any impairing substance; alcohol was positive in 24.4% and THC in 2.7% of drivers. A 2018 Italian study^27^ of 1026 injured drivers found that 30.6% tested positive for an impairing substance; 17.3% had BAC greater than 0.05%, 13.6% tested positive for a medicinal drug, and 1.5% had THC greater than 2 ng/mL. These studies used different sampling strategies, which limits direct comparison of results. Nevertheless, like the current research, all report a high prevalence of substance use, especially alcohol, in injured drivers. The higher prevalence of THC in North American drivers vs European or Australian drivers may reflect differences in cannabis consumption rates, in traffic policy and enforcement, and/or in drug use and driving cultures.

We found marked geographic variation in the prevalence of substance use. Prevalence was lowest in British Columbia and highest in Atlantic Canada. This pattern, which persisted after adjustment for driver age, driver sex, and crash type, may be explained by differences in driving and substance use culture, differences in social acceptability of driving after substance use, differences in enforcement of traffic laws, and different prescribing practices (for opioids, depressants, and/or medical cannabis). We also found that substance use was more frequent in drivers with a rural vs an urban address. Other researchers also found higher rates of alcohol and/or drug use in rural drivers,^28^ which is likely due to multiple factors such as less access to public transportation or taxis in rural areas, difficulty enforcing traffic laws in rural areas, and differences in driving culture and risk perception.^29^

The current study found variation in substance use by age. Compared with middle-aged drivers (45-55 years), drivers younger than 19 years had a lower prevalence of all substances except THC; they were less likely to test positive for any impairing substance and less likely to have BAC greater than 0.08%. These lower rates are probably explained by zero-tolerance laws for alcohol or drug use in novice drivers. Unfortunately, 7.6% of young drivers had BAC greater than 0.08%. This finding contrasts with Canadian roadside surveys, which found almost no alcohol use in drivers younger than 19 years.^8^ This discrepancy likely reflects the extreme collision risk in young drinking drivers^30^ which results in them being over-represented in crash statistics. In contrast with alcohol, THC was twice as prevalent in drivers younger than 19 years compared with drivers aged 45 to 54 years, likely because many young drivers believe it is safe to drive after using cannabis and that the risk of being caught by police is low.^31^ Moreover, cannabis is predominantly a recreational drug of adolescents and younger adults.^32^ These findings suggest the need for increased enforcement of zero-tolerance laws in novice drivers, combined with public education on the risks of alcohol and/or cannabis in young drivers.

The use of most substances was highest in drivers aged 19 to 44 years. Alcohol use was highest in drivers aged 25 to 34 years, suggesting increasing alcohol use once drivers are no longer subject to zero-tolerance laws. THC, especially at higher levels, was most common in drivers aged 19 to 24 years, followed by drivers aged 25 to 34 years. The highest rate of stimulant use was in drivers aged 25 to 44 years. These findings suggest the need for increased enforcement measures and social marketing campaigns targeting alcohol, cannabis, and recreational drug use in young to middle-aged drivers.

Older drivers were less likely to use substances with the exception of opioids and depressants. Opioids were most often detected in drivers aged 35 to 74 years and depressants in drivers aged 65 years or older, likely reflecting greater use of prescription medications in older drivers. Use of depressants and opioids by older drivers is concerning because their sedative effects likely exacerbate the impact of age-related declines in reaction time, vision, and cognition. These findings speak to the importance of minimizing sedating medication use in older drivers combined with education on the risks of sedating medications.

The current study also found that more males used recreational substances (alcohol, cannabis, and stimulants) whereas more females used depressants, including prescription medications used to treat anxiety, depression, or other mental health problems. These findings are consistent with the epidemiology of mental health disorders: women have higher rates of anxiety and mood disorders than men, whereas men have higher rates of substance use disorders.^33^

### Strengths and Limitations

Our study had several strengths. Blood tests were obtained to guide trauma management, and we conducted comprehensive toxicology testing on all drivers for whom excess blood was available. This process minimized the selection bias that would occur if toxicology tests were ordered based on clinical suspicion of drug use. Further, our design permitted ethical review board approval for waiver of consent, which allowed us to avoid the refusal bias that could occur if consent were required and drivers who used drugs or alcohol were less likely to participate. We included drivers treated in 15 trauma centers in 8 provinces, making our results more generalizable than if we included only a few hospitals. Data were collected prospectively over a 4-year period, which mitigates the risk of having results skewed by transient events that temporarily impact substance use.

This study also had limitations. Our methods did not allow us to interview or examine drivers. As such, we do not know when the driver used an impairing substance, the route of administration, or if they were impaired. This limitation also means that we were unable to study factors such as impulsivity, risk taking behavior, and anxiety or mood disorders, which also contribute to substance use.^34,35^ Unavailability of excess blood in many drivers may be a concern, but we believe that this factor is unlikely to have caused bias because availability of excess blood is mostly related to hospital laboratory processes and not driver characteristics. Our findings may not apply to minor-injury collisions that do not require blood tests. The study period included cases from before, during, and after the COVID-19 pandemic, which may have impacted drug and alcohol use in some drivers.

## Conclusions

More than one-half of the drivers in this national cross-sectional study tested positive for an impairing substance and more than one-fifth had used multiple classes of substances. THC was detected slightly more often than alcohol, although alcohol remains the greater threat to road safety based on levels in excess of regulations and high collision risk. Prevalence for most substances was higher in younger drivers and in males, while older drivers and females were more likely to have used depressants. Moreover, while highest in Atlantic Canada, the prevalence of substance use was high across the country. These findings suggest that impaired driving remains a substantial road safety concern in Canada. Continued monitoring is required to develop rational targeted interventions and to evaluate the effectiveness of prevention measures.

## Notes

Supplement 1. **eTable 1.** Substances Included in Toxicology Analysis **eTable 2.** Additional Collision Characteristics by Region **eTable 3.** Substance Prevalence Among Injured Drivers According to Other Risk Factors **eTable 4.** Crude Prevalence (%) and 95% Confidence Intervals (One-Sample Proportion Assuming Asymptotic Normality) by Region **eFigure.** Crude Prevalence (%) for Substance Use by Region **eTable 5.** Adjusted Odds Ratios (aORs) and 95% Confidence Intervals (CIs) from Adjusted Logistic Regression Models for Substance Use

Supplement 2.
Data Sharing Statement
