# Supplementary material for: Prevalence of Impairing Substance Use in Injured Drivers
Source: JAMA Netw Open. 2025 Apr 22;8(4):e256379. doi: 10.1001/jamanetworkopen.2025.6379 (PMC12015673; doi:10.1001/jamanetworkopen.2025.6379)
Supplement: Supplement 1. — eTable 1. Substances Included in Toxicology Analysis eTable 2. Additional Collision Characteristics by Region eTable 3. Substance Prevalence Among Injured Drivers According to Other Risk Factors eTable 4. Crude Prevalence (%) and 95% Confidence Intervals (One-Sample Proportion Assuming Asymptotic Normality) by Region eFigure. Crude Prevalence (%) for Substance Use by Region eTable 5. Adjusted Odds Ratios (aORs) and 95% Confidence Intervals (CIs) from Adjusted Logistic Regression Models for Substance Use [file jamanetwopen-e256379-s001.pdf]

Supplemental Online Content

Brubacher JR, Erdelyi S, Chan H, et al. Prevalence of impairing substance use in injured drivers. *JAMA Netw. Open*. 2025;8(4):e256379. doi:10.1001/jamanetworkopen.2025.6379

eTable 1. Substances Included in Toxicology Analysis

eTable 2. Additional Collision Characteristics by Region

eTable 3. Substance Prevalence Among Injured Drivers According to Other Risk Factors

eTable 4. Crude Prevalence (%) and 95% Confidence Intervals (One-Sample Proportion Assuming Asymptotic Normality) by Region

eFigure. Crude Prevalence (%) for Substance Use by Region

eTable 5. Adjusted Odds Ratios (aORs) and 95% Confidence Intervals (CIs) from Adjusted Logistic Regression Models for Substance Use

This supplemental material has been provided by the authors to give readers additional information about their work.

© 2025 Brubacher JR et al. *JAMA Network Open*.

eTable 1. Substances Included in Toxicology Analysis

Category	Details
Alcohol*	Alcohol (Ethanol)
Cannabinoids*	THC
Opioids*	6-Acetylmorphine (indicates heroin use), Buprenorphine, Codeine, EDDP (methadone metabolite), Fentanyl, Hydrocodone, Hydromorphone, Meperidine, Methadone, Mitragynine (found in "kratom"; opioid properties), Morphine, Norfentanyl, Oxycodone, Tramadol
Stimulants*	Amphetamine, Benzoylecgonine (cocaine metabolite), Cocaethylene (cocaine metabolite), Cocaine, MDA, MDMA, Methamphetamine
Depressants*	
Benzodiazepines	7-Aminoclonazepam, 7-Aminoflunitrazepam, 7-Aminonitrazepam, Alprazolam, Chlordiazepoxide, Chlorpheniramine , Clonazepam, Diazepam, Etizolam, Flunitrazepam, Lorazepam, Midazolam, Nitrazepam, Nordiazepam, Oxazepam, Temazepam, Zolpidem, Zopiclone
Antidepressants	Amitriptyline, Bupropion, Citalopram, Clomipramine, Desipramine, Doxepin, Fluoxetine, Hydroxybupropion, Imipramine, Mirtazapine, didesmethylcitalopram, Norcitalopram, Norsertraline, Nortriptyline, O-Desmethylvenlafaxine, Paroxetine, Sertraline, Trazodone, Venlafaxine
Anticonvulsants	Carbamazepine, Cyclobenzaprine, Lamotrigine, Phenobarbital, Phenytoin, Topiramate
Antipsychotics	Chlorpromazine, Clozapine, Haloperidol, Hydroxyrisperidone, Loxapine, Olanzapine, Quetiapine, Risperidone, Ziprasidone, Zuclopenthixol
Antihistamines	Cetirizine, Cyclobenzaprine, Diphenhydramine, Doxylamine, Tripelennamine
Miscellaneous depressants	Gabapentin, Dextromethorphan (found in OTC cold and cough preparations; dissociative properties in high doses)

* "Any substance" includes detection of at least one of Alcohol, THC, Opioids, Stimulants, or Depressants.
"Polysubstance" includes detection of at least two of Alcohol, THC, Opioids, Stimulants, or Depressants.

© 2025 Brubacher JR et al. *JAMA Network Open*.

eTable 2. Additional Collision Characteristics by Region [count (percent)]

	National	British Columbia	Alberta	Saskatchewan	Ontario	Quebec	Atlantic Provinces
Number of drivers	8328 (100%)	2418 (100%)	1605 (100%)	577 (100%)	1823 (100%)	1281 (100%)	624 (100%)
Crash time of day							
06:01-10:00	1264 (15.2%)	378 (15.6%)	251 (15.6%)	89 (15.4%)	276 (15.1%)	189 (14.8%)	81 (13.0%)
10:01-14:00	1609 (19.3%)	500 (20.7%)	333 (20.7%)	88 (15.3%)	304 (16.7%)	258 (20.1%)	126 (20.2%)
14:01-18:00	2261 (27.1%)	698 (28.9%)	426 (26.5%)	145 (25.1%)	451 (24.7%)	352 (27.5%)	189 (30.3%)
18:01-22:00	1615 (19.4%)	456 (18.9%)	330 (20.6%)	122 (21.1%)	349 (19.1%)	254 (19.8%)	104 (16.7%)
22:01-02:00	966 (11.6%)	243 (10.0%)	171 (10.7%)	63 (10.9%)	284 (15.6%)	130 (10.1%)	75 (12.0%)
02:01-06:00	567 (6.8%)	140 (5.8%)	94 (5.9%)	70 (12.1%)	127 (7.0%)	89 (6.9%)	47 (7.5%)
Missing	46 (0.6%)	3 (0.1%)	0 (0%)	0 (0%)	32 (1.8%)	9 (0.7%)	2 (0.3%)
Crash day of week							
Weekday	5369 (64.5%)	1618 (66.9%)	1104 (68.8%)	361 (62.6%)	1099 (60.3%)	804 (62.8%)	383 (61.4%)
Weekend of holiday	2959 (35.5%)	800 (33.1%)	501 (31.2%)	216 (37.4%)	724 (39.7%)	477 (37.2%)	241 (38.6%)
Crash year							
2019	2228 (26.8%)	755 (31.2%)	678 (42.2%)	157 (27.2%)	515 (28.3%)	60 (4.7%)	63 (10.1%)
2020	1157 (13.9%)	315 (13.0%)	223 (13.9%)	109 (18.9%)	126 (6.9%)	247 (19.3%)	137 (22.0%)
2021	2461 (29.6%)	706 (29.2%)	383 (23.9%)	152 (26.3%)	454 (24.9%)	461 (36.0%)	305 (48.9%)
2022	2030 (24.4%)	449 (18.6%)	321 (20.0%)	122 (21.1%)	585 (32.1%)	434 (33.9%)	119 (19.1%)
2023	452 (5.4%)	193 (8.0%)	0 (0%)	37 (6.4%)	143 (7.8%)	79 (6.2%)	0 (0%)
Crash season							
Winter	1838 (22.1%)	490 (20.3%)	366 (22.8%)	120 (20.8%)	408 (22.4%)	333 (26.0%)	121 (19.4%)
Spring	1886 (22.6%)	618 (25.6%)	351 (21.9%)	148 (25.6%)	388 (21.3%)	257 (20.1%)	124 (19.9%)
Summer	2380 (28.6%)	669 (27.7%)	466 (29.0%)	156 (27.0%)	543 (29.8%)	333 (26.0%)	213 (34.1%)
Fall	2224 (26.7%)	641 (26.5%)	422 (26.3%)	153 (26.5%)	484 (26.5%)	358 (27.9%)	166 (26.6%)

© 2025 Brubacher JR et al. *JAMA Network Open*.

eTable 3. Substance Prevalence [count (percent)] Among Injured Drivers According to Other Risk Factors

	BAC > 0	BAC ≥ 0.08%	THC > 0	THC ≥ 5 ng/mL	Stimulants	Depressants	Opioids	Any substance	Poly substance
National (all drivers)	1341 (16.1%)	1024 (12.3%)	1354 (16.3%)	277 (3.3%)	1057 (12.7%)	2368 (28.4%)	905 (10.9%)	4568 (54.9%)	1798 (21.6%)
Injury severity									
Admitted	643 (21.2%)	476 (15.7%)	549 (18.1%)	104 (3.4%)	452 (14.9%)	919 (30.2%)	445 (14.6%)	1884 (62.0%)	802 (26.4%)
Treated and released	696 (13.2%)	547 (10.4%)	803 (15.2%)	173 (3.3%)	604 (11.4%)	1447 (27.4%)	460 (8.7%)	2680 (50.8%)	993 (18.8%)
Crash type									
Single-vehicle	936 (27.7%)	740 (21.9%)	669 (19.8%)	149 (4.4%)	621 (18.4%)	1073 (31.8%)	468 (13.9%)	2239 (66.4%)	1069 (31.7%)
Multi-vehicle	404 (8.2%)	283 (5.7%)	685 (13.8%)	128 (2.6%)	435 (8.8%)	1294 (26.1%)	437 (8.8%)	2327 (47.0%)	728 (14.7%)
Crash time of day									
06:01-10:00	104 (8.2%)	73 (5.8%)	182 (14.4%)	36 (2.8%)	155 (12.3%)	338 (26.7%)	124 (9.8%)	612 (48.4%)	225 (17.8%)
10:01-14:00	100 (6.2%)	60 (3.7%)	216 (13.4%)	48 (3.0%)	171 (10.6%)	516 (32.1%)	172 (10.7%)	808 (50.2%)	273 (17.0%)
14:01-18:00	208 (9.2%)	143 (6.3%)	345 (15.3%)	69 (3.1%)	235 (10.4%)	673 (29.8%)	236 (10.4%)	1172 (51.8%)	394 (17.4%)
18:01-22:00	337 (20.9%)	243 (15.0%)	280 (17.3%)	65 (4.0%)	210 (13.0%)	433 (26.8%)	185 (11.5%)	915 (56.7%)	392 (24.3%)
22:01-02:00	340 (35.2%)	281 (29.1%)	181 (18.7%)	36 (3.7%)	158 (16.4%)	245 (25.4%)	123 (12.7%)	628 (65.0%)	297 (30.7%)
02:01-06:00	239 (42.2%)	214 (37.7%)	138 (24.3%)	22 (3.9%)	122 (21.5%)	149 (26.3%)	59 (10.4%)	400 (70.5%)	202 (35.6%)
Crash day of week									
Weekday	628 (11.7%)	476 (8.9%)	785 (14.6%)	165 (3.1%)	628 (11.7%)	1565 (29.1%)	584 (10.9%)	2813 (52.4%)	1018 (19.0%)
Weekend or holiday	713 (24.1%)	548 (18.5%)	569 (19.2%)	112 (3.8%)	429 (14.5%)	803 (27.1%)	321 (10.8%)	1755 (59.3%)	780 (26.4%)
Crash year									
2019	343 (25.6%)	273 (26.7%)	435 (32.1%)	85 (30.7%)	239 (22.6%)	644 (27.2%)	258 (28.5%)	1263 (27.6%)	486 (27.0%)
2020	188 (14.0%)	138 (13.5%)	191 (14.1%)	54 (19.5%)	176 (16.7%)	318 (13.4%)	161 (17.8%)	633 (13.9%)	275 (15.3%)
2021	444 (33.1%)	338 (33.0%)	433 (32.0%)	86 (31.0%)	332 (31.4%)	704 (29.7%)	275 (30.4%)	1360 (29.8%)	602 (33.5%)
2022	307 (22.9%)	232 (22.7%)	244 (18.0%)	46 (16.6%)	252 (23.8%)	586 (24.7%)	175 (19.3%)	1085 (23.8%)	364 (20.2%)
2023	59 (4.4%)	43 (4.2%)	51 (3.8%)	6 (2.2%)	58 (5.5%)	116 (4.9%)	36 (4.0%)	227 (5.0%)	71 (3.9%)
Crash season									
Winter	244 (18.2%)	195 (19.0%)	271 (20.0%)	51 (18.4%)	203 (19.2%)	520 (22.0%)	184 (20.3%)	956 (20.9%)	359 (20.0%)
Spring	336 (25.1%)	259 (25.3%)	311 (23.0%)	63 (22.7%)	233 (22.0%)	538 (22.7%)	191 (21.1%)	1038 (22.7%)	417 (23.2%)
Summer	438 (32.7%)	318 (31.1%)	397 (29.3%)	74 (26.7%)	311 (29.4%)	677 (28.6%)	255 (28.2%)	1350 (29.6%)	522 (29.0%)
Fall	323 (24.1%)	252 (24.6%)	375 (27.7%)	89 (32.1%)	310 (29.3%)	633 (26.7%)	275 (30.4%)	1224 (26.8%)	500 (27.8%)

© 2025 Brubacher JR et al. *JAMA Network Open*.

eTable 4. Crude Prevalence (%) and 95% Confidence Intervals (One-Sample Proportion Assuming Asymptotic Normality) by Region

	British Columbia	Alberta	Saskatchewan	Ontario	Quebec	Atlantic provinces
BAC > 0	11.2 (10.0, 12.6)	14.4 (12.7, 16.2)	20.3 (17.1, 23.8)	18.8 (17.0, 20.6)	18.9 (16.8, 21.2)	22.0 (18.8, 25.5)
BAC ≥ 0.08%	8.4 (7.4, 9.6)	11.8 (10.3, 13.5)	14.7 (12.0, 17.9)	15.0 (13.4, 16.7)	13.2 (11.4, 15.2)	16.5 (13.7, 19.7)
THC > 0	15.6 (14.2, 17.1)	12.8 (11.2, 14.5)	19.1 (16.0, 22.6)	13.4 (11.9, 15.1)	19.8 (17.6, 22.1)	26.3 (22.9, 30.0)
THC ≥ 5 ng/mL	2.9 (2.2, 3.6)	3.1 (2.3, 4.0)	5.4 (3.7, 7.6)	1.9 (1.4, 2.7)	3.7 (2.8, 5.0)	7.2 (5.4, 9.6)
Stimulants	10.9 (9.7, 12.2)	13.3 (11.7, 15.1)	12.3 (9.8, 15.3)	11.8 (10.4, 13.4)	13.6 (11.8, 15.6)	19.1 (16.1, 22.4)
Depressants	24.9 (23.2, 26.6)	31.0 (28.7, 33.3)	31.5 (27.8, 35.5)	25.2 (23.3, 27.3)	28.4 (26.0, 31.0)	42.3 (38.4, 46.3)
Opioids	8.4 (7.3, 9.6)	15.8 (14.0, 17.7)	13.9 (11.2, 17.0)	9.8 (8.5, 11.2)	8.1 (6.7, 9.8)	13.9 (11.4, 17.0)
Any substance	48.5 (46.5, 50.5)	57.5 (55.0, 59.9)	61.4 (57.2, 65.3)	52.3 (50.0, 54.6)	57.1 (54.4, 59.9)	69.6 (65.7, 73.1)
Poly substance	17.2 (15.8, 18.8)	22.7 (20.7, 24.8)	25.1 (21.7, 28.9)	19.6 (17.9, 21.6)	22.7 (20.5, 25.1)	35.7 (32.0, 39.7)

© 2025 Brubacher JR et al. *JAMA Network Open*.

eFigure. Crude Prevalence (%) for Substance Use by Region

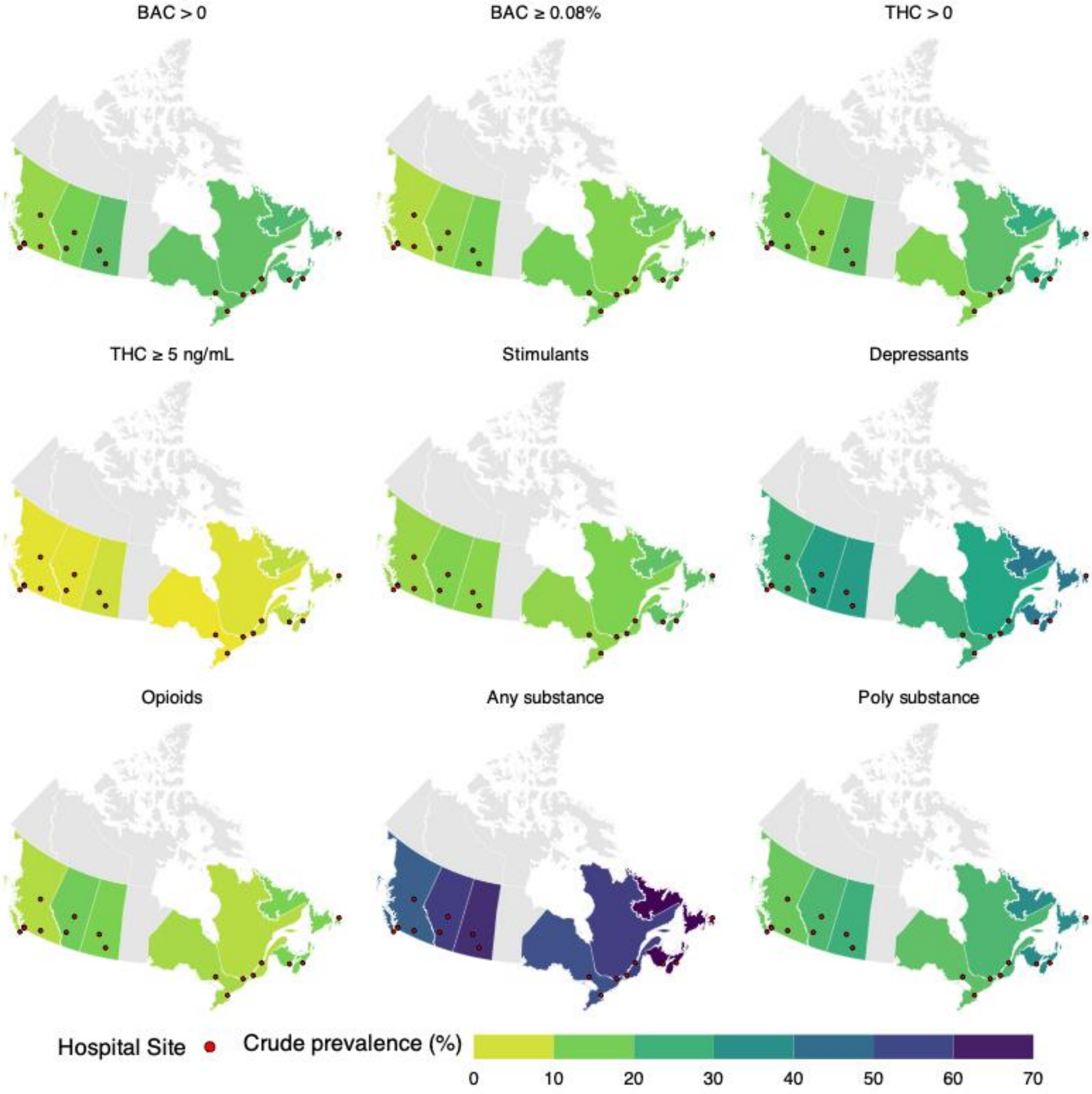

© 2025 Brubacher JR et al. *JAMA Network Open*.

eTable 5. Adjusted Odds Ratios (aORs) and 95% Confidence Intervals (CIs) from Adjusted Logistic Regression Models for Substance Use

P-values reported for each risk factor were computed based on a Wald-Chi-square test with cluster robust standard errors.

	BAC > 0	BAC ≥ 0.08%	THC > 0	THC ≥ 5 ng/mL	CNS stimulants	CNS depressants	Opioids	Any substance	Poly substance
	Wald-Chi-square P-value, aOR (95%CI)								
Demographics									
**Region**	**p<0.001**	**p=0.31**	**p<0.001**	**p=0.11**	**p=0.001**	**p<0.001**	**p<0.001**	**p<0.001**	**p<0.001**
British Columbia	1.00 (ref)	1.00 (ref)	1.00 (ref)	1.00 (ref)	1.00 (ref)	1.00 (ref)	1.00 (ref)	1.00 (ref)	1.00 (ref)
Alberta	1.16 (0.73, 1.84)	1.28 (0.95, 1.72)	0.69 (0.33, 1.42)	0.94 (0.37, 2.34)	1.21 (0.73, 2.00)	1.32 (0.96, 1.81)	1.94 (1.36, 2.78)	1.30 (0.90, 1.90)	1.27 (0.84, 1.93)
Saskatchewan	1.19 (0.75, 1.91)	1.12 (0.73, 1.72)	0.91 (0.63, 1.30)	1.55 (0.78, 3.09)	0.85 (0.56, 1.27)	1.20 (0.87, 1.65)	1.40 (0.62, 3.14)	1.22 (0.86, 1.72)	0.97 (0.68, 1.37)
Ontario	1.30 (0.81, 2.09)	1.38 (1.05, 1.82)	0.73 (0.60, 0.88)	0.63 (0.19, 2.04)	0.94 (0.58, 1.51)	0.99 (0.74, 1.33)	0.98 (0.77, 1.24)	0.92 (0.66, 1.28)	0.93 (0.65, 1.33)
Quebec	1.60 (0.99, 2.57)	1.40 (1.03, 1.90)	1.37 (0.90, 2.09)	1.31 (0.84, 2.04)	1.10 (0.54, 2.23)	1.18 (0.71, 1.97)	0.85 (0.56, 1.29)	1.29 (0.86, 1.95)	1.21 (0.74, 1.97)
Atlantic provinces	1.50 (0.96, 2.35)	1.40 (0.86, 2.28)	1.68 (1.32, 2.13)	2.24 (1.21, 4.18)	1.46 (0.89, 2.41)	2.17 (1.45, 3.26)	1.36 (0.95, 1.94)	1.98 (1.30, 3.02)	1.93 (1.22, 3.07)
**Age group (years)**	**p<0.001**	**p<0.001**	**p<0.001**	**p<0.001**	**p<0.001**	**p<0.001**	**p<0.001**	**p<0.001**	**p<0.001**
<19	0.58 (0.34, 0.98)	0.38 (0.22, 0.66)	2.01 (1.49, 2.71)	2.53 (1.19, 5.37)	0.48 (0.26, 0.89)	0.46 (0.34, 0.60)	0.31 (0.14, 0.71)	0.66 (0.49, 0.90)	0.50 (0.32, 0.76)
19-24	1.31 (1.06, 1.61)	1.26 (1.02, 1.54)	2.79 (2.32, 3.36)	4.69 (2.76, 7.98)	0.93 (0.68, 1.27)	0.56 (0.45, 0.70)	0.63 (0.44, 0.91)	1.10 (0.94, 1.28)	1.02 (0.85, 1.24)
25-34	1.59 (1.33, 1.90)	1.57 (1.28, 1.93)	2.23 (1.88, 2.64)	3.70 (2.30, 5.94)	1.52 (1.18, 1.96)	0.70 (0.58, 0.86)	0.76 (0.63, 0.92)	1.31 (1.13, 1.51)	1.32 (1.10, 1.59)
35-44	1.25 (1.02, 1.53)	1.27 (1.01, 1.60)	1.36 (1.07, 1.73)	2.50 (1.28, 4.90)	1.66 (1.33, 2.06)	0.92 (0.78, 1.10)	1.05 (0.86, 1.29)	1.30 (1.16, 1.46)	1.34 (1.11, 1.60)
45-54	1.00 (ref)	1.00 (ref)	1.00 (ref)	1.00 (ref)	1.00 (ref)	1.00 (ref)	1.00 (ref)	1.00 (ref)	1.00 (ref)
55-64	0.77 (0.60, 0.99)	0.69 (0.51, 0.94)	0.83 (0.70, 0.98)	1.28 (0.82, 2.00)	0.57 (0.46, 0.72)	1.01 (0.84, 1.21)	1.01 (0.84, 1.23)	0.90 (0.79, 1.02)	0.85 (0.75, 0.97)
65-74	0.51 (0.36, 0.73)	0.30 (0.20, 0.46)	0.45 (0.38, 0.53)	1.15 (0.50, 2.61)	0.18 (0.09, 0.36)	1.24 (1.01, 1.53)	0.94 (0.78, 1.14)	0.90 (0.73, 1.10)	0.55 (0.42, 0.72)
>74	0.23 (0.12, 0.42)	0.13 (0.05, 0.38)	0.27 (0.13, 0.57)	0.35 (0.07, 1.67)	0.21 (0.14, 0.32)	1.07 (0.91, 1.27)	0.47 (0.32, 0.70)	0.68 (0.56, 0.82)	0.30 (0.20, 0.47)
**Sex**	**p<0.001**	**p=0.02**	**p<0.001**	**p<0.001**	**p<0.001**	**p<0.001**	**p=0.38**	**p=0.57**	**p=0.09**
Male	1.53 (1.21, 1.92)	1.35 (1.06, 1.72)	1.66 (1.48, 1.86)	1.78 (1.33, 2.40)	1.53 (1.34, 1.75)	0.54 (0.47, 0.62)	1.08 (0.91, 1.29)	0.97 (0.87, 1.08)	1.11 (0.99, 1.26)
Female	1.00 (ref)	1.00 (ref)	1.00 (ref)	1.00 (ref)	1.00 (ref)	1.00 (ref)	1.00 (ref)	1.00 (ref)	1.00 (ref)
**Residential postal code**	**p<0.001**	**p<0.001**	**p=0.16**	**p=0.48**	**p=0.03**	**p=0.003**	**p=0.003**	**p<0.001**	**p<0.001**
Urban	1.00 (ref)	1.00 (ref)	1.00 (ref)	1.00 (ref)	1.00 (ref)	1.00 (ref)	1.00 (ref)	1.00 (ref)	1.00 (ref)
Rural	1.51 (1.29, 1.76)	1.49 (1.34, 1.66)	1.19 (0.94, 1.52)	0.80 (0.44, 1.47)	1.32 (1.03, 1.70)	1.28 (1.09, 1.51)	1.26 (1.08, 1.47)	1.40 (1.20, 1.63)	1.55 (1.23, 1.95)
Collision characteristics									
**Injury severity**	**p<0.001**	**p=0.003**	**p=0.001**	**p=0.30**	**p=0.002**	**p=0.13**	**p<0.001**	**p<0.001**	**p<0.001**
Admitted	1.50 (1.22, 1.85)	1.37 (1.11, 1.69)	1.22 (1.08, 1.38)	1.12 (0.90, 1.39)	1.30 (1.10, 1.54)	1.12 (0.97, 1.30)	1.78 (1.49, 2.12)	1.50 (1.26, 1.78)	1.44 (1.24, 1.66)
Treated and released	1.00 (ref)	1.00 (ref)	1.00 (ref)	1.00 (ref)	1.00 (ref)	1.00 (ref)	1.00 (ref)	1.00 (ref)	1.00 (ref)
**Crash type**	**p<0.001**	**p<0.001**	**p<0.001**	**p<0.001**	**p<0.001**	**p<0.001**	**p<0.001**	**p<0.001**	**p<0.001**
Single-vehicle	3.13 (2.49, 3.95)	3.36 (2.50, 4.50)	1.27 (1.14, 1.41)	1.44 (1.16, 1.78)	2.03 (1.76, 2.35)	1.44 (1.28, 1.62)	1.55 (1.27, 1.91)	1.90 (1.63, 2.22)	2.24 (1.91, 2.63)
Multi-vehicle	1.00 (ref)	1.00 (ref)	1.00 (ref)	1.00 (ref)	1.00 (ref)	1.00 (ref)	1.00 (ref)	1.00 (ref)	1.00 (ref)
**Crash time of day**	**p<0.001**	**p<0.001**	**p=0.76**	**p=0.36**	**p<0.001**	**p=0.03**	**p=0.08**	**p<0.001**	**p<0.001**
06:01-10:00	1.25 (0.88, 1.78)	1.38 (0.87, 2.19)	0.96 (0.71, 1.30)	0.87 (0.53, 1.44)	1.03 (0.80, 1.32)	0.79 (0.68, 0.93)	0.94 (0.72, 1.23)	0.91 (0.78, 1.05)	0.98 (0.81, 1.19)
10:01-14:00	1.00 (ref)	1.00 (ref)	1.00 (ref)	1.00 (ref)	1.00 (ref)	1.00 (ref)	1.00 (ref)	1.00 (ref)	1.00 (ref)
14:01-18:00	1.40 (1.09, 1.80)	1.54 (1.14, 2.08)	1.04 (0.87, 1.26)	0.95 (0.61, 1.49)	0.88 (0.71, 1.10)	0.93 (0.84, 1.04)	0.99 (0.75, 1.32)	1.04 (0.92, 1.18)	0.97 (0.84, 1.14)
18:01-22:00	2.96 (2.35, 3.72)	3.22 (2.40, 4.32)	1.01 (0.81, 1.26)	1.03 (0.69, 1.55)	0.95 (0.77, 1.17)	0.84 (0.75, 0.94)	1.02 (0.82, 1.27)	1.13 (0.99, 1.28)	1.25 (1.07, 1.45)
22:01-02:00	5.75 (4.37, 7.55)	6.89 (5.04, 9.41)	0.97 (0.79, 1.18)	0.85 (0.39, 1.84)	1.09 (0.83, 1.42)	0.86 (0.69, 1.06)	1.22 (1.01, 1.47)	1.54 (1.38, 1.73)	1.65 (1.46, 1.86)
02:01-06:00	5.71 (4.01, 8.12)	7.69 (4.79, 12.34)	1.14 (0.92, 1.41)	0.71 (0.37, 1.38)	1.40 (1.12, 1.74)	0.85 (0.67, 1.07)	0.85 (0.63, 1.14)	1.67 (1.30, 2.15)	1.63 (1.30, 2.03)
**Crash day of week**	**p<0.001**	**p<0.001**	**p=0.02**	**p=0.64**	**p=0.41**	**p=0.18**	**p=0.69**	**p=0.003**	**p=0.01**
Weekday	1.00 (ref)	1.00 (ref)	1.00 (ref)	1.00 (ref)	1.00 (ref)	1.00 (ref)	1.00 (ref)	1.00 (ref)	1.00 (ref)
Weekend of holiday	1.77 (1.51, 2.08)	1.72 (1.43, 2.06)	1.20 (1.02, 1.41)	1.08 (0.80, 1.45)	1.08 (0.89, 1.32)	0.92 (0.82, 1.04)	0.97 (0.86, 1.10)	1.13 (1.04, 1.23)	1.28 (1.05, 1.56)
**Crash year**	**p=0.10**	**p=0.04**	**p<0.001**	**p=0.05**	**p<0.001**	**p=0.20**	**p<0.001**	**p=0.35**	**p<0.001**
2019	0.88 (0.69, 1.12)	0.93 (0.66, 1.31)	1.52 (1.29, 1.79)	1.02 (0.68, 1.53)	0.68 (0.52, 0.88)	1.19 (0.90, 1.57)	0.73 (0.50, 1.06)	1.18 (0.97, 1.44)	0.92 (0.74, 1.15)
2020	1.00 (ref)	1.00 (ref)	1.00 (ref)	1.00 (ref)	1.00 (ref)	1.00 (ref)	1.00 (ref)	1.00 (ref)	1.00 (ref)
2021	1.03 (0.81, 1.32)	1.09 (0.76, 1.58)	1.07 (0.77, 1.49)	0.78 (0.46, 1.34)	0.86 (0.69, 1.07)	1.11 (0.92, 1.35)	0.79 (0.65, 0.97)	1.04 (0.82, 1.32)	1.03 (0.83, 1.29)
2022	0.81 (0.58, 1.12)	0.84 (0.55, 1.28)	0.74 (0.49, 1.12)	0.55 (0.34, 0.88)	0.83 (0.63, 1.11)	1.19 (1.02, 1.39)	0.61 (0.50, 0.74)	1.02 (0.85, 1.22)	0.73 (0.60, 0.89)
2023	0.87 (0.59, 1.29)	0.83 (0.50, 1.38)	0.75 (0.44, 1.25)	0.39 (0.12, 1.22)	1.12 (0.73, 1.72)	1.13 (0.69, 1.85)	0.79 (0.59, 1.05)	1.13 (0.78, 1.63)	0.80 (0.59, 1.08)
**Crash season**	**p=0.002**	**p=0.15**	**p=0.75**	**p=0.45**	**p=0.17**	**p=1.00**	**p<0.001**	**p=0.88**	**p=0.35**
Winter	0.77 (0.56, 1.05)	0.84 (0.60, 1.18)	0.95 (0.73, 1.22)	0.87 (0.64, 1.19)	0.94 (0.76, 1.16)	1.01 (0.86, 1.20)	1.00 (0.75, 1.34)	0.96 (0.82, 1.13)	0.93 (0.77, 1.12)
Spring	1.00 (ref)	1.00 (ref)	1.00 (ref)	1.00 (ref)	1.00 (ref)	1.00 (ref)	1.00 (ref)	1.00 (ref)	1.00 (ref)
Summer	0.89 (0.76, 1.05)	0.83 (0.69, 1.00)	0.90 (0.73, 1.10)	0.84 (0.63, 1.13)	1.04 (0.87, 1.26)	1.01 (0.82, 1.24)	0.97 (0.77, 1.23)	1.02 (0.84, 1.23)	0.87 (0.75, 1.01)
Fall	0.72 (0.59, 0.86)	0.75 (0.56, 1.00)	0.94 (0.74, 1.19)	1.06 (0.81, 1.38)	1.17 (0.96, 1.43)	1.02 (0.85, 1.22)	1.22 (0.97, 1.52)	1.02 (0.85, 1.23)	0.95 (0.76, 1.20)

© 2025 Brubacher JR et al. *JAMA Network Open*.
