# Supplementary material for: Prevalence of Impairing Substance Use in Injured Drivers
Source: JAMA Netw Open. 2025 Apr 22;8(4):e256379. doi: 10.1001/jamanetworkopen.2025.6379 (PMC12015673; doi:10.1001/jamanetworkopen.2025.6379)
Supplement: Supplement 2. — Data Sharing Statement [file jamanetwopen-e256379-s002.pdf]

Data Sharing Statement

Brubacher. Prevalence of Impairing Substance Use in Injured Drivers. *JAMA Netw Open*. Published April 22, 2025. doi:10.1001/jamanetworkopen.2025.6379

Data
Data available: No

Additional Information
Explanation for why data not available: Potentially sensitive data; sharing not allowed per REB approved protocol
